# Automation in analytical chemistry—from rule of thumb to fully automated methods. Some philosophies and social consequences

**DOI:** 10.1155/S1463924684000031

**Published:** 1984

**Authors:** R. Stanley

**Affiliations:** 1 Department of History of Science and Technology University of Manchester Institute of Science and Technology PO Box 88 Manchester M60 1QD UK; 2 16 Newbury Grove Hopwood Lancashire Heywood 0LI0 2PL UK


Journal of Automatic Chemistry, Volume 6, Number 1 (January–March 1984), pages 6–13

### Automation in analytical chemistry—from rule of thumb to fully automated methods. Some philosophies and social consequences

**R. Stanley**

*Department of History of Science and Technology, University of Manchester Institute of Science and Technology, PO Box 88, Manchester M60 1QD, UK**

### Historical brief

It seems a far cry from 1761 when the chemist's place of work was defined in the following way: 'Laboratory or Elaboratory, the chemists workhouse or the place where they perform their operations, where their furnaces are built, their vessels kept, etc. and in general the term laboratory is applied to any place where physical experiments in pharmacy, chemistry, pyrotechny etc. are performed' [1]. By 1880 it was pointed out to the American Society of Microscopists that 'the better the instrument the more reliable the results obtained, and the best work cannot be done even by the most expert worker except with the best instruments' [2]. Of course we have seen dramatic changes in the appearance of analytical instruments since those days, and by the mid 1950s George D. Beal could confidently state that the analytical laboratory was, in his estimation, 'the Supreme Court of the chemical profession in its relation to industry' [3].

From early times, equipment for chemical transformations has been made by man—his machines having been evolved from apparatus of the kitchen, the pharmacy and the forge. Quality and purity measurements became fairly widespread, the only really qualitative tests being those for the assay of gold and silver by cupellation. The 17th century witnessed many new instruments, such as the telescope, microscope, air pump and pneumatic trough for the collection of gases. Eighteenth-century preoccupations with fire paved the way for the introduction of the mouth blowpipe, which further extended the range of analytical chemistry. Hydrometers, burettes, pipettes, polarimeters, viscometers, bunsen burners, colorimeters, and a whole host of varied and important instruments became available to the laboratory during the 18th and 19th centuries.

The 19th century was a period of consolidation. Chemical analysis grew into an art, with a sound empirical basis involving weight relationships and chemical combinations. Techniques on gravimetric analysis were continually refined and there emerged new concepts of physical chemistry towards the turn of the century. Techniques in volumetric analysis were gradually improved, though it only became effective in industrial applications around the middle of the century, and it matured as an accurate method of analysis with the advent of sensitive indicators and the general acceptance of titrimetric procedures. The first titrimeric method was probably carried out by Claude Joseph Geoffroy in 1729 when he showed that Orleans' vinegar was stronger than the Parisian variety [4]. Descroizilles described a burette for the volumetric determination of alkaline materials in 1806.

Instrumental methods at the turn of the century were well received. They had an important role to play in industrial research and a greater speed and sensitivity in repeated analyses more than justified initial costs. The theory of analytical chemistry became more refined towards the end of the 19th century, and physical chemistry became popular through scientists such as Wilhelm Ostwald (1853–1932), William Gibb (1839–1905) and Wilhelm Pfeffer (1845–1920). Theoretical titrimetry became established, as did electrometric analysis—Robert Behrend (1856–1926) performed the first potentiometric titration in 1893.

Twentieth-century analytical chemistry had a firm footing, both theoretical and practical, on which to build techniques in the future. Theory and practice were becoming cemented together with the aids of better laboratories, improved tools of experimentation and a more coherent approach to the problems in analytical chemistry, though analytical methods remained similar to 19th-century techniques until the emergence of physical methods in applied electricity. The Laboratory of the Government Chemist in Clements Inn Passage, London, was originally equipped in 1895 and it represented a significant landmark in analytical workshops in Britain because of its advanced design. It set a precedent for others and was a showpiece of the late Victorian age [5]. The benches had gas controls, sinks and waste-disposal facilities which were planned for accessibility when servicing. Other features were a centrally-heated water supply (which served as a hot plate as well as a drying cupboard), a vacuum line, a microscope, a balance, a flash-point apparatus, a refractometer, a polarimeter and a centrifuge for milk analysis. The Government Chemist's laboratory was in no way a reflection of most contemporary laboratories. From around the turn of the century until about the 1930s, analytical laboratories were furnished with equipment typical of that used for traditional analyses and such wet analytical techniques dominated procedures used in analysing the materials of production and research well into the 30s when electrically powered aids became more commonplace. Analytical chemistry then was 'a rather unspectacular handmaiden of the other fields of chemistry.... Some researchers employed micro methods, particularly in organic analysis, and

* *Correspondence to 16 Newbury Grove, Hopwood, Heywood, Lancashire OL10 2PL, UK.*

unusual organic reagents, occasionally were used to detect and estimate trace amounts of particular ions' [6]. Spectrochemical analysis had been developed initially during the latter years of the 19th century and the technique began to be used by many industrial and research establishments. Colorimetric analysis in particular was a widespread technique using the now obsolete Duboscq Colorimeter.

Though classical wet chemical methods were the dominant processes used to analyse the materials of commerce, micro-analysis emerged as a branch of science through chemists such as Friedrich Emich (1869–1940) and Fritz Pregl (1860–1930). Many techniques appeared or were perfected in the early 20th century. Gravimetric analysis, thermal analysis, titrimetric analysis, non-aqueous acid base titrimetry, non-aqueous redox titrimetry, chelometric and complexiometric methods, functional group analysis and kinetic methods of analysis all represent the kind of impetus that was given in the field of analytical chemistry.

### The Influences of the World Wars

The First World War 'greatly accelerated the demand for many things and resulted in a large increase in industrial production' [7]. New and improved products created the need for careful process-control requirements—classical methods of analysis could hardly cope. At the end of the War there was a further steady increase in production. New elements were finding their way into new products which rendered the older methods of analysis inadequate. The improved methods of production accentuated the requirements for more trained research chemists to undertake more precise and faster analytical techniques. Laws, theories, factual information and instruments gathered in the fields of chemistry, physics and other related subjects. These converging ideas enabled analytical chemistry to emerge from the era of the burette and balance and into the era of the spectrometer and titrimeter—the instrumental era [8]. 1930 marked the first significant use of electronic techniques in analytical chemistry. Various devices such as photo-electric systems had been tried, but the recording spectrophotometer was the first successful instrument. It was invented by A. C. Hardy at Massachusetts Institute of Technology and it incorporated a thyatron, which energized a pen motor and drove a polarizer, which restored null conditions in a Hüfner-type photometer. Since 1930, the phenomenal growth in electronics has resulted in a major revolution in analytical instrumentation. The rapid development of the vacuum-tube amplifier and the photo-electric tube, and more recently, of transistors and other semi-conductor devices, has resulted in the establishment of many analytical methods based upon them.

The Second World War placed heavy demands on the chemical industry for explosives, light metals, synthetic rubber, quality aviation fuels, synthetic oils, fats, medicinals and purified isotopes. In the USA, the chemical industry grew enormously. Despite their subjection to heavy bombing, the Germans had produced the V-2 missile which required its own special fuel. The end of hostilities resulted in a Cold War, which, in turn, created demands for nuclear research. There were other areas of research such as in jet propulsion and rocket fuels. Investigations into outer space drew attention to elements and compounds that had formerly been a mere curiosity. In 1957, B. W. Bradford and D. L. Nicholson of Imperial Chemical Industries illustrated only too well how the emergence of new and improved materials, which had appeared as a result of Wartime hostilities, created a demand for improved methods of analysis during the period 1947–1957 [9].

The most striking development up to the early 1960s was concerned with electro-analytical methods—major facets of which included potentiometry, polarography, voltametry, conductiometry and coulometry. With the introduction of the manufacture of spectrometers covering the ultra-violet, visible and infra-red ranges, spectroscopic methods in analytical chemistry were promoted to a prestigious level as an analytical tool. Spectrographic methods depending on the measurement of the wavelength and intensity of light emitted by an element or compound in a flame or an electric arc have replaced some of the more conventional methods of qualitative analysis. An innovatory and revolutionary analytical tool which was widely used after 1950—chromatography—completely altered the approach to problems associated with the separation and analysis of mixtures of organic and inorganic compounds. Absorption, partition, ion exchange and vapour-phase chromatography all became popular techniques. Analysis incorporating the use of radioactive tracers were exhaustively tried and was implemented following the availability of radioactive isotopes. Micro-chemical methods using milligram quantities produced extremely accurate results and had profound influence on the rate of progress in organic chemistry. Methods of analysis were available in the early 1960s for the determination of most elements occurring in organic compounds, and determination of oxygen became possible in 1947. The use of improved electronic devices became popular and resulted in greater sophistication of analytical methods: this enabled analytical results to be displayed by pen-recording devices, which subsequently enabled analytical methods to be carried out completely automatically.

### The processing and interpretation of analytical data

The most dramatic growth in signal processing and control over the past quarter of a century has come about in the field of digital electronics. By 1948, experiments were already in hand in connection with stored program digital computers, but by the time these first-generation computers were completed in the early 1950s, they were already obsolete. Advanced knowledge about the subject of discrete solid-state electronics progressed rapidly; and, by the late 1950s, specialization in large digital computers was such that they could be used in scientific applications. Initially such machines were used to handle massive numerical calculations. By the mid 1960s, chemists were becoming enthusiastic about the application of the dedicated computer. In the UK the situation appeared to be very similar to that in the USA, and dramatic changes in analytical procedures were very apparent between the years 1954 and 1957 in the field of process control [10]. In 1954, Walter J. Murphy (editor of *Analytical Chemistry*) prophesied the way ahead in analytical chemistry with a fair degree of accuracy as to what it might have promised to society at large [11]. In contemplating the future in terms of the instrumental regime of analytical chemistry Magnus Pyke noted:

> Traditionally the analyst has prized his technical dexterity at the bench. Yet there are today clear signs for all who are prepared to read them that increasingly we shall find analytical techniques being carried out automatically. Already there are automatic spectrographs for steel analysis, automatic analysis assemblies for determining dissolved oxygen designed by the Water Pollution Board, and even general purpose automatic analysis equipment commercially available for those who wish to arrange for routine operations to be done by machine rather than by shift chemist. As this kind of apparatus becomes more

> common, clearly skill and dexterity in the laboratory arts will be less in demand [12].

The signs were there for all to see, the classical chemist was to feature less and less in the field of analytical control as the art and science of analytical control by instrumentation advanced. The chemist trained in the rigours of classical analytical chemistry would have to change his whole outlook towards these newer procedures of analysis, or become obsolete during a period when the economic growth of an industrial (or otherwise) organization was becoming largely dependent upon paying low wages to its employees. Fully automatic procedures would, of course, increasingly feature in analytical procedures, even though it was appreciated that these systems had a high initial cost. In the long term they can be proved to be less labour intensive and capable of being used in a profitable manner. The impact of instrumental techniques upon industry was perhaps best described by G. F. Smith in 1957:

> Fantastic applications and accompanying instrumentation as applied to analytical operations have served a major role in glamourising the field. This is the major influence in the ascendancy of analytical research and routine industrial control in the last quarter century. The click of the Geiger counter, the flick of the neon-button indicators, the whine of the centrifuge, the graphic recording of the Brown chart and the fabulous simplicity of the operations of gas-phase chromatography are but casual examples. The multiplicity of electronically operated instrumentation devices has been the basis of a rapidly growing industry devoted exclusively to supplying the necessary equipment. The requisite monetary outlay involved seems to be no deterrent. From the electron microscope to the mass spectrometer, and all the intermediate installations, instrumentation serves the field without noticeable retardation [13].

From about the end of the Second World War to the mid 1950s, three factors appear to have been operating which gave added impetus to the growth and popularity of instrumentation and automation of analytical processes. These factors may be seen as antecedents of the complex factors operating today:

(1) A decline in manpower available for analytical work.
(2) A rapid increase in the volume of analytical work required for the control of new plants.
(3) A very marked increase in the complexity of new analytical problems arising from the development of new products, particularly in the organic field.

### The trends towards automation

As instruments and systems became more sophisticated, they were further refined as a result of advances made in computerized techniques and data handling. Different systems began to be interfaced and miniaturization of electronic gadgetry allowed such systems to be featured in more compact forms. The revolution in the field of electronics has perhaps had the most far-reaching consequences for automated analysis. A divisional chief analyst [14] of a research department of a large industrial chemical complex isolated some of the growth areas in instrumentation within his company during the period 1955–1980. This particular research department then dealt with some 15 000 samples per annum. The period between 1960 and 1965 was thought to be the rapid growth time which resulted from the use of mechanical means of automation such as syringe pumps, crozet motors, cam timers, syringe dispensers and analogue displays. Between 1968 and 1975 was thought to be the period when the newer techniques of analysis were appearing and it was the start of the laboratory computing era. Analytical problems were becoming more complex. These years represent the era of the microprocessor. Although there is now a proliferation of fairly sophisticated equipment and computers, their use depends on the number of samples and the frequency of presentation and whether the analysis of batches is possible. Much can be gained from partial automation and very often a demand for analysis grows with analytical capability. As the employment of analytical manpower has become more and more expensive, the decline in the number of analytical chemists has been a direct function of the use of analysis instruments. There has been a transitional phase from bench analysis to laboratory instrumentation [15]. The ultimate in laboratory automation, which has in some cases already been achieved, can be thought of as the extensive replacement of laboratory analytical control by continuously operating process stream quality-control instruments which are used to direct integrated automatic-control systems. The use of semi- and fully automated techniques went a long way to solving problems associated with the limited manpower problems—problems that J. Craik had noted in 1957 when he was Chairman of the ICI Nobel Division [15].

### Where have all the chemists gone?

The period 1950–1960s witnessed the decline of the chemist [16]—where before older instruments had been used by experienced analysts almost as a sideline, newer generation analysts took over, all too often with very little or no knowledge of chemistry. The classical chemist was to feature less and less in industrial laboratories, and this situation was to deteriorate rapidly to the point where there was an acute shortage of such people. Wartime experiences had made the government re-evaluate education in Britain because hostilities had made the country even more aware of the fact that it depended upon scientifically and technologically trained staff. If Britain was to achieve its manpower targets set in science and technology as outlined by the Percy Report on Higher Technological Education, and the Barlow Report of 1946, it was thought that university output would have to double [17]. This doubling of the number of scientists and technologists did in fact come about. Consistent with such expansion, a trend of students staying on for sixth form education prior to university entrance came about. Many further-education institutions acquired full status of universities. The expansion in scientific subjects was also stimulated by interests in jet propulsion. Industry could not get the requisite number of such trained scientists to work in their laboratories. The economy was running at almost full employment, and demands for industrial scientists, chemists, electrical and mechanical engineers remained in excess of supply from about the late 1940s to the middle 1950s. Such factors contributed in no small way to the shortage of suitably qualified analysts. Moreover, subjects such as mathematics and statistics, electronics and physics were beginning to attract students who otherwise might have become analytical chemists. With incentives in other academic disciplines, with analytical chemistry being held in poor esteem, and a 'creaming off' to university of good practical and academic laboratory staff, the use of semi- and fully automated systems in analytical quality control became a vital necessity. Another deleterious factor aggravated this situation during the 1960s, which must have been working contrary to trends that would otherwise have been beneficial to industry in Britain. There was a serious drift abroad of scientists and technologists which gave rise to the catch phrase—'The Brain Drain' [18]. It is now more than apparent that the era of the classical chemist is over, and events have justified the prophecies made in the past. Heavy investment in American space programmes has resulted in the emergence of specialized analytical equipment that is capable of sampling and analysing on the surfaces of far-off planets. Indeed, it has been some of these kinds of ventures in the USA that have lured British top scientists and technologists abroad. The skills of the analyst can never be totally superseded for his knowledge and applications were and are still needed for the analysis of standards used in the setting up, calibration and control of instruments. The qualified chemist still retains a vital role as the arbiter between customer and supplier, and this role is governed by procedures which use established, standardized classical methods of analysis which were developed over many years. Albeit, certainly in the UK, it might be fair comment to state that analytical chemistry as a profession has become downgraded by a complex matrix of factors outlined above and possibly some others not mentioned here. Objectively, the analyst became regarded by many as representing an overhead charge to production, and his skills, accuracy, reproducibility, efficiency and fatigue effects could, in many cases, be bettered by instruments of this ever-expanding 'black-box' era.

### Trends towards full automation

In the USA there appears to have been a more dynamic attitude to the full utilization of automation in analytical chemistry when contrasted against the situation in the UK. By 1958, the analyst was becoming more challenged by the advancement of technology with the inherent advantages offered by mechanized instruments [19]. As noted already, the analyst was regarded as representing a high overhead charge to production. It became more important for the chemist to think in terms of cost/man-hour or pennies/test. Analysis for its own sake was seen as an objective that was no longer valid. Ultimate precision in analytical processes was seen not to be a prime requirement of the chemist, as in most cases an error of $\pm 5\%$ would not normally be of too much significance. Analytical speed became an important criterion in analytical control, and complete analysis in many ways was seen as giving a pointer to the efficient operation of a process. As instrumental procedures began to be applied to analytical control, their operators were not always required to have a knowledge of the chemistry of a particular process or to know the chemistry of the reactions involved which dictated the choice of technique for analysis, and hence the instrumentation being utilized. Only when analytical results became abnormal were questions asked about their implications. The overriding factors which prescribed the analytical procedures used was the time occupied in each control test, and the wage of the individual worker. Such a situation was well illustrated by L. H. Lampitt of the Lyons Laboratories, London, in 1952 at the International Congress on Analytical Chemistry [20]. More unqualified labour began to be used in analytical control as instrumentation and automation began to feature more in production laboratories. However, it was well appreciated that, when operated by the most careful of technicians, such sophisticated instruments could not replace the experience of the analytical chemist. Such chemists qualified in the more traditional techniques were becoming more of a rarity by 1960 [21]. As a consequence, the use of continuous analysers for plant control was in many cases thought to be of paramount importance. The situation was much the same in the USA in 1963, and attempts were being made to attract qualified graduates into instrument-development groups. Such a shortage of qualified personnel was a problem apparently shared by the whole instrument industry [22].

More and more literature on instrumental techniques appeared in textbooks as analysts grappled with the bewildering proliferation of new machines. The preface to A. I. Vogel's third edition of *Qualitative Inorganic Analysis* (1961) indicated the importance that he thought should be placed upon the extended use of instrumental analysis: 'It is generally accepted that the training in at least some of the instrumental methods is highly desirable' [23]. Included in the text are detailed descriptions and applications of various instruments. This standard reference work presents a balance between classical and instrumental techniques and also gives due emphasis to the requirements of older techniques needed for standardizing instrumental procedures. By today's standards many of the instruments illustrated by Vogel could be 'relegated to the attic or the cellar', but the theoretical principles upon which many of these instruments operated (or perhaps still do operate) still apply to the more streamlined machines of today with their multifunctional capabilities. Vogel's book in many ways reflects the transitional stage from the truly classical procedures to those instrumental methods which would ultimately merge into fully automated techniques. At the time of printing, Vogel noted that, due to limitations on space, topics such as X-ray methods, mass spectroscopy, gas chromatography, radiochemical methods, nuclear magnetic resonance spectroscopy, polarimetry and refractometry had to be omitted from the book. These omissions alone illustrate the vast number of instruments that could then be acquired.

Naturally enough, with such increases in the use of physical methods of analysis, problems did arise in connection with training in instrumental methods [24]. Many of the interconnected problems associated with recruitment are still with us, and it remains fashionable for companies to recruit laboratory assistants with minimal or no qualifications at all, which can be a financial advantage in the long term.

### Changing economies

The 1950s witnessed increasing demands in 'money wages' following the events of the Second World War. In the USA, real compensation per man-hour in the private economy has risen faster than productivity since 1940. During the early 1960s, real hourly earnings increased less than productivity, and unit costs were stable for the period. Between 1966 and 1971, real earnings and money earnings had exceeded productivity gains, and this resulted in a sharp rise in unit labour costs—especially in the 1970s. Between 1947 and 1970, the output per man-hour in the total private economy showed an annual increase of 3·2% compared with the average increase of 5·1% in the compensation per man-hour. In 1970, annual increases in productivity fell to 0·9%, whilst compensation per man-hour rose by 7%.

Following the War, Great Britain evolved an incomes policy with a view to restraining wages and prices, which, it was hoped, would compensate for the relatively slow growth in productivity. It was thought necessary to maintain a viable international trade balance. Both Labour and Conservative governments inaugurated incomes policies—Labour's 'wage freeze' between 1948 and 1950, and the Tory 'wage pause' between 1961 and 1962. Other measures were implemented to resolve the worsening economic situation. The National Board of Prices and Incomes was terminated in 1971. A wages explosion occurred in early 1970 before elections returned the Conservatives to power, with earnings increasing by 15% during the year. Other developments up to 1973 occurred to aggravate the British economy [25]. The economic boom of 1973 was brought to an abrupt halt in 1974 by the oil crisis, which caused recession and accelerated inflation. The effects on wages and employment were dramatic and this was reflected in a decrease in the hours of working [26].

Consistent with such inflationary trends in the cost of materials, professionally trained and well-qualified analytical chemists became expensive commodities at a time when low paid and unskilled technicians could achieve better and faster analytical results by using sophisticated instrumentation. Such set-ups offered greater flexibility, were less prone to fatigue effects and were found to be more cost-effective than human beings. The trend began when instrumental techniques began to be applied to analytical processes, and it has carried on unabated since with the effect that laboratories of the future may well be operated by neon-flashing robots that do not incorporate the instinctive correction facilities that were so often found inherently in the dedicated and experienced analyst.

### The changing face of industrial analytical laboratories

Analysis is fundamental to chemical operations. A chemical synthesis can only be considered to be complete when the product has been fully analysed and its component parts determined. Analysis is the common language of the chemist in industry. As effective analysis has become more important in the face of economic constraint and a requirement for close specification control, it has come to rely even more on instrumentation and automation. Analysis is the basis of national and international commercial agreements which must depend on internationally agreed methods and interpretations. Analysis in industry embraces overlapping aspects of the overall objectives of analytical control: research, process development and process control. As increased demands were placed on analysts in industry, central laboratories introduced instruments that saved on mundane laboratory tasks and improved speed. Simpler analytical instruments were available for use on the plant so that shop-floor personnel could undertake their own determinations to get stream composition values. This eliminated the sample 'lag time' existing between the laboratory and the plant. Monitoring instruments were introduced, which directly recorded various critical compositions from plant streams. Manual analysis was becoming replaced more by mechanical aids or the use of devices that could measure directly a physical property related to chemical composition. Mechanized machines included automatic and semi-automatic titrimeters, automatic apparatus to determine thermal arrest points for such purposes as crystallizing and freezing, automatic distillation apparatus for determining the Reid vapour pressure of petrol. Techniques used for measurement of a physical property included absorption and emission spectrophotometry, mass spectrometry, gas chromatography and an assortment of now household names in the field of such sophisticated techniques. Even by 1957 analysts were confronted by a bewildering choice of instruments that were available for off-line and in-line applications. Many systems that were commercially available were of American origin, and this is still the case today. Though there was a proliferation of machines to control operating plants, in many cases there was a scarcity of reliable, quality-controlled instruments. This was in part due to a lack of confidence at the interface between the instrument user and the instrument manufacturer—a situation that seemed to be more pronounced in Britain than it was in the USA. A comprehensive review of the instruments available for industry was given in 1964 [27]; American machines were particularly featured. The situation with regard to the instrument business in the UK has changed little from the early 1960s to date [28]. Such instruments were so sophisticated that in many cases they were capable of performing the majority of analyses that could be made in the plant-control laboratory in suitable circumstances. Most on-line analysers around in the mid 1960s were expensive—typically between about £1000 and £3500. Not only were more American instruments available, but they were used more extensively in the USA [31]—one American firm with 20 chromatographs thought itself to be 'lagging behind'; another, was apparently using 100 on-line chromatographs (half for automatic control) in petrochemical applications. A similar situation existed in German firms. It seems ironical that by 1950 the British instrument business had apparently established itself with high standards and it was apparently well advanced [32]. Many parameters seem to have contributed to Britain's declining role in this industry—a less flamboyant attitude, a poor attention to communications and applications at the interface of analytical science and production technique, ill-documented literature, a lack of suitably trained staff, a cynical attitude to overall instrument credibility, poor investment in laboratory equipment [33], a poor awareness of the technical developments of mechanical aids for research and development, poor applications engineering, poor salesmanship as a result of lack of technical knowledge, unfavourable tax incentives, the 'Brain Drain', the effects of industrial and government secrecy as a legacy of War [34, 35 and 36], and a lack of desire to interface different technologies that contribute to the instrument business.

Such factors have all played their part, and some universities in the UK have recently set up departments that bring together separate skills and disciplines and interface theoretical matters derived from other areas, such as physics, mathematics, computer science and management science. From this has emerged a recognizable discipline of analytical science, which embraces the sociological implications brought about by the use of microelectronics. By becoming involved in the multi-disciplinary approach this newly recognized analytical format the analyst ideally becomes an integral part of a team who take collective responsibility for a production process, and his new technology should provide the automated means of finding meaningful results. P. B. Stockwell succinctly defines automation as 'a multifaceted and multidimensional problem, the various aspects of which are fully interlocking a multidisciplinary problem' [37]—the total systems approach. The skills required to achieve effective automation include electronics, statistics, computer expertise, with an associated knowledge of business and organizational skills. Perhaps Britain is at last 'getting it together'.

The 1974 oil crisis escalated the costs of labour generally and the costs associated with employing analytical staff began to take an even greater slice of company profits. With the emergence of multi-processor technology, computer facilities came to be adopted more in analytical control, particularly because they could be applied to dedicated and flexible analytical systems. Such machines had the ability to handle even more complex analytical data. Computerization is now a necessity in the field of analytical instrumentation and it has come of age in being able to interface modularized analytical systems. This in turn has extended analytical capabilities and has led to many improvements in economic performance on the industrial front. From communications [38] with a large number of chemical firms and allied manufacturing industries in the UK, it was apparent that many of these organizations began to adopt analytical facilities with 'state-of-the-art' electronics from about the mid 1970s—particularly those large competitive industries that could afford them. It is interesting to note that from analysis of such communications with a variety of chemical companies, that the period between about 1964–1973 was a time when companies were buying even fewer instruments than they had been in the decade 1950–1960 when labour costs were not what they were to become during the 1970s. The worsening economic situation from about 1974 became more apparent to industrialists who quickly realized that computer-interfaced systems could realistically become very economical and therefore help to cut down labour costs. This trend of upgrading analytical facilities has become widespread, although the use of such modern instrumentation does not necessarily apply to all analytical strategies—particularly where a batch operation is used as the normal production mode.

Automation of continuous processes has led to the formulation of generalized theories, philosophies, definitions and new applications appertaining to chemical processes at the control, and research and development stage. An overview of the state of 'Instrumentation and control in process industries' in 1973 [39] indicated that the adoption of computer-controlled processes was delayed by a lack of knowledge about particular processes and the theoretical variables involved, and the lack of instrumentation for continuously monitoring some of the process variables.

D. S. Harder in 1936—being employed by General Motors Corporation defined automation as 'the automatic handling of parts between progressive production processes'. A definition of automation given in *Encyclopaedia Britannica* in 1973 [40] was a little more comprehensive: the term 'automation' evolved outside the disciplines of analytical chemistry and may not always be appropriate in that context. In a 1968 thesis [41], D. G. Porter of the Laboratory of the Government Chemist surveyed the various definitions that have been put forward in relation to laboratory automation by workers such as R. Jonnard (1960) who defined it as 'the science concerned with the techniques, methods and principles involved in each phase of qualitative data acquisition, reduction, conversion, transmission, storage, retrieving, handling and computation by automatic instrumentation'. Porter outlined the distinction between mechanization, instrumentation and automation in laboratory applications. The term 'automation', accepted in 1958, was considered to be a 'relatively new word in the lexicon of analysts' [42]. A generalized definition of automation was given by R. Kuzel *et al.* [43] in 1969; as early as 1957, Gorden D. Patterson Jr. had suggested a broader definition of automation that would not only be applicable in process or manufacturing operations [44]. A rationalized definition of automatic analysis which embraces most of the techniques available to analytical chemistry was given by Foreman and Stockwell in 1975 [45] who quoted from the Commission of Analytical Nomenclature of the Analytical Division of the International Union of Pure and Applied Chemistry. They also gave a definition for mechanization. Whatever definition is used to describe fast analytical methods, it is clear that they have a vital part to play in chemical industries. Foreman and Stockwell note that expansion in the field of automated analysis is characterized in our standards of living and general well-being [46], and that such instrumentation provides an important analytical service to industry where analytical information can be produced quickly and evaluated rapidly. In industry, such analytical procedures may be accomplished off-line, on-line, or in-line with a relatively low total analysis time. Continuous-flow and discrete sample-processing systems have been exploited successfully in a variety of configuations in both research and industrial laboratories. A review in 1969 by Kuzel *et al.* [47] gives details of the various automated and semi-automated systems then available, and they also give extensive references to other work in the field at that time. The paper also highlights the advantages of using automated uniformity, a greater optimization of analyst's time, labour savings, increased laboratory throughput, an early warning of production and process difficulties, and a speedier transfer from research and development programmes to full-scale production. On the negative side, automation can be ineffective on a small number of samples where a variety of products is processed, where the method does not comply with official compendia, where the technique does not lend itself to automation, or, perhaps more important, where the costing of automatic equipment may be greater than any anticipated saving. There are now well-established philosophies concerning automation and there are also some futuristic ideas as to where automation and computing are leading to [48]. Whether just mechanized, partly automated or fully automated, a proliferation of sophisticated instrumentation is described in scientific literature. The changes that have occurred as a result of the microprocessor revolution have been dramatic in the very recent past. A complete world-wide listing of scientific equipment manufacturers, dealers and distributors appeared in the *Laboratory Buyer's Guide Edition* of *International Laboratory* in 1980 and it contains a great deal of information that cannot be gained elsewhere. Its objectives are to aid laboratory researchers in obtaining information about products available—highlighting an awareness of the need to communicate ideas across a multitude of disciplines which lend themselves to some form of interfacing in the broadest sense of the word. The edition has since appeared annually. An analysis of the international companies listed in the 1980 edition illustrates that the USA still has by far the largest commitment to laboratory management [49].

### The present status of automation in analytical chemistry

It has become increasingly apparent that automated procedures have become an essential element of analytical control in the laboratory, and their use is not restricted to clinical laboratories and process control. Automation can involve all stages of an analytical procedure including sampling, sample preparation, separation, standardization, calibration, data collection and processing, reporting results and data storage and retrieval. Over the past 30 or more years chemistry has expanded tremendously, as witnessed by the logarithmic increase in the associated journals. In no small measure, it has been due to the innovation and adaptation for common use of instrumental techniques. Like other branches of the subject, chemical analysis has benefited and the subject has been transformed from that based on chemical technique to one based on physical methods. Consequently, concepts and philosophies have been introduced into analysis which even a few years ago might have been purely associated with other disciplines. The modern-day analyst needs to develop skills and familiarize himself with the theoretical matters derived from physics, mathematics, electronics, computer science, and technology and management science. Crystallizing out from the interfacing of these multidisciplines has been a recognizable discipline of analytical science which further embraces wider horizons other than those bounded by analytical chemistry. In recognizing the importance and implications of analytical science, the Department of Chemistry at the University College of Swansea, introduced a BSc in the subject, and the Swansea Summer School of Automatic Chemical Analysis was conceived within this framework. First given in

1979, the Summer School was received with enthusiasm. In 1980 the School was equally, if not more successful, and like the previous and all subsequent meetings, it drew lecturers of international repute who detailed the latest thinking on all aspects of chemical analysis. Like the analytical department at Swansea, a similar department has been established at the University of Manchester Institute of Science and Technology to look into the applications of automated analytical techniques at a high academic level under the guidance of three appointed professors.

There is now a wealth of information scattered in the literature which describes automatic equipment and its associated gadgetry which can be used in analytical control and research. The appearance of the *Journal of Automatic Chemistry* represents 'the beginning of the end: the end of a struggle to legitimize automation in chemistry and the beginning of what', Dr Peter B. Stockwell says, 'will be a valuable means of communication for those of us as chemists, who become increasingly involved with automatic analysis'.

### Some generalized conclusions

Much talent has been applied in the service of analytical chemistry and its application to industry, and there has been profuse practical applications of technology to production over many years. Multi-disciplinary expertise required by the chemical industry has encompassed the talents of chemists, engineers, physicists, biologists and managers. Chemical analysis is concerned with the utilization of diagnostic chemical action on small- and large-scale industrial processes that depend on chemical principles. Journals relating to the state-of-the-art of analytical chemistry began to appear and such publications proliferated as greater importance began to be placed on this subject, which was becoming transformed from rule-of-thumb techniques to a specific discipline in its own right. As it gradually became more obvious that chemical manufacture was based on scientific principles and that knowledge of such principles was important in terms of industrial competition, it was realized that there would be a need for increased technical or educated skill and a wider diffusion of the knowledge of scientific principles. Such a climate permitted the assayer to emerge from his mysterious alchemical workshop to develop his imaginative and diagnostic talents in modern laboratories surrounded by a host of ultra-modern, dehumanized and automated aids. Chemical production has considerable potential for causing environmental damage, and there sometimes exists antagonism between energy requirements and material goods on the one hand, and environmental pollution on the other. Angus Smith—the famous Alkali Inspector over 100 years ago almost single-handedly created a style of surveillance over industrial pollution which has become a tradition. His talents and application allowed pollution not merely to become minimized, but to become optimized—to strike a balance between the benefits of productive industry and clean air. Since that time, various analytical procedures have developed for different manufacturing industries which have extended the practice of analytical chemistry and illuminated malpractices. As the chemical industry grew, so did a new set of analytical requirements for chemical control of the processes involved for economic and social reasons—not merely to curtail the effects of pollution and chemical adulteration—but to determine the quality of raw materials and finished products from industrial manufacture whose products were becoming more specialized. As such developments became apparent, chemical apparatus became refined and more accurate, and new analytical techniques developed into more complex procedures.

After the two World Wars, it became apparent that standards of living generally would improve, and there was a requirement to mass produce a whole range of consumer products which required an even greater degree of quality control. As a result, instrumental analytical procedures developed which furnished industrialists with the means of fully optimizing processes in terms of decreased product losses, formulations, efficiency, process material recovery, continuous monitoring, energy conservation, product quality, lower recycling costs, and other related aspects of efficient industrial production. Many of these precise production techniques were closely guarded in an age of commercial confidentiality which was rife following the end of the Second War, and instrumental techniques in many cases were developed shrouded by secrecy at a time when the theoretical aspects of electricity were being rigorously applied to instrumental systems. At the same time it became apparent that there was a shortage of analytical chemists, technicians and laboratory assistants. This trend is still apparent today and is similar inasmuch that the cost of labour is expensive and its amortization in the long term is poor compared to sophisticated instruments costing many thousands of pounds. Analytical chemists as independent arbiters give technical judgements and often give rise to public disaffection at the academic and industrial level.

Often, parallels are drawn between scientific and technical research and economic well-being. Ambiguity here occurs by economic recession in the West and political oppression around the world, and only too often there are widespread fears that new applications of such technology, whilst raising standards of living, may threaten collective security. In such a climate educated issues are raised as specialized syllabi and heavy emphasis placed upon academically biassed examination requirements has made wider the social and professional tiers between those with academic and those with practical skills, and may have also made worse the socio-economic divisions. Many teachers in Britain are not involved in the conditions of the market place in which their students must find employment. Such a condition can be said to have contributed to a widespread ignorance of the ways in which much classroom knowledge may be relevant to the industrial world. A more coherent interfacing of curricula should be apparent through the levels of science and social education from the primary through to the tertiary levels. Moveover, the educator should be prepared to become re-educated to keep abreast of new technologies as they affect the interfacing of his discipline with industry and the social and economic world in which we live, and the penetrating implications that the world of microprocessor technology has to offer. Related to this is the desperate shortage of graduate science teachers at the secondary level and the poor career structure. A trend in the early 1970s was that many scientists moved into posts such as accountancy where basic numeracy and basic logic are important. They liked what they observed and they stayed. Growth was also apparent on the numerate and social sciences such as business studies, management sciences, accounting and financial management. In our rapidly changing technological society, changes are taking place more than at any time in our history. Now adaptability and wide-ranging personal skills are required and just being a good scientist is not enough (perhaps it never was). Scientists need to be 'interfaced' at all levels to embrace the full consequences of the processes they set out to control and develop.

In recent times, it may be said that in Britain, the ambitious student often became a victim of the 'dirty-hands disease'—a situation whereby he quite easily became infatuated with those academic courses promising better financial rewards and greater personal recognition. There has been a reaction against the popularity of subjects such as analytical science in recent times, even though such subjects can be said to be the backbone of the economic prosperity of this country. The application of automatic control to analytical chemistry is a typical example of how Britain has the available technology but failed to capitalize upon the principles involved. This situation was typified by the intuitive exploits of John Sargrove, a Londoner, who died in 1974 and whose work is now almost entirely forgotten. He and his team of 20 young engineers laid down the foundations for modern automation which was seen as the answer to post-War labour shortages. From 1947 to 1949 the team designed 'master brains' to automate industrial processes; he was a victim of political pressure and tried to convince people that automation would not create unemployment—rather, that it meant re-deployment. His arguments did not prevail; money dried up and so did government interest. Now, 30 years later, the UK has to import electronic circuit-making equipment similar to that made by Sargrove [50]. Like automation in analytical chemistry, this is a doleful reminder of how the UK fails to capitalize on technical ideas. In the 19th century, the British chemical industry failed to exploit Perkins's discoveries. Foreign instrument competitors very often seem to have much better selling organizations, highly trained staffs of technical scientists ready to help any customer with instrumental problems, and a much more outward-looking attitude to business generally. Over the last few decades in Britain, there appears to have been lacking a willingness to bring together more the instrument maker and the instrument user. Applications engineering likewise has not been exploited to the full. In Britain, male models appear to rank higher than engineers. It has been said that Britain's current poor economic performance may be blamed on the predominantly non-technical backgrounds from which the senior decision-makers in British industry, finance and government are drawn. The attitude of the instrument user to the instrument maker has waxed and waned throughout time, and the lack of rapport between the two appears to have been more pronounced in the UK. Perhaps it has always been reflected in the class position held by the humble craftsmen and the snobbish intellectuals, and this again is perhaps reflected in their academic choice of subjects is already noted. The USA does not seem to have such deep-rooted intellectual rivalries, and, typical of its instrument companies, Technicon Instrument Corporation were able to capitalize on interfacing a whole variety of disciplines to fully exploit the instrumentation business. For instance, they surmounted the problems of professional secrecy by inviting professionals to contribute to their manuals, publications and international symposia.

Liebig drew parallels between the prosperity of a nation and the quantity of sulphuric acid it produced. Perhaps a more intrinsic study of analytical and control instruments would reveal similar conclusions. Dr Lyon Playfair in the 19th century was only too well aware of the systematic industrial training of qualified students in industrial studies in Germany in his report on industrial construction on the Continent in 1852. This situation is somehow reminiscent of the situation prevailing in the UK engineering industry, and has parallels in the instrument business in the UK, as Sir Monty Finniston said in his report *Engineering Our Future, 1980*:

> Inventive talents have not been harnessed effectively by manufacturing industry because, compared with Continental Europe and the large part of the world which has followed its lead, there have been neither the pecuniary rewards in this country to attract sufficient of the brightest national talents into engineering in industry...

### References

1. *Encyclopaedia Britannica*, **2**, 857.
2. Blackman, G. E., *Buffalo Meeting of American Society of Microscopists* (1880).
3. Beal, George, D., *Analytical Chemistry*, **26** (1954), 794.
4. Madsen, Rancke E., *The Development of Titrimetric Analysis till 1806* (Copenhagen, 1958), 25.
5. Lewis, D. T., *Chemistry and Industry* (1963), 1705.
6. Ihde, Aaron, J., *The Development of Modern Chemistry* (New York, 1964), 559.
7. Willard, Hobart, H., *Analytical Chemistry*, **23**, 1727.
8. Ibid., 1728.
9. Bradford, B. W. and Nicholson, D. L., in *Proceedings of the Congress on Modern Analytical Chemistry in Industry* (1957), 161.
10. Pyke, Magnus, in *Proceedings of the Congress on Modern Analytical Chemistry in Industry*, ibid., 1.
11. Murphy, Walter, J., *Analytical Chemistry*, **26** (1954), 945.
12. Pyke, op. cit.
13. Smith, G. Frederick, in *Proceedings of the Congress on Modern Analytical Chemistry in Industry*, op. cit., 170.
14. Interview (1980).
15. Bradford and Nicholson, op. cit., 165.
16. Warburton, D., Production control analysis in copper based alloys (a paper presented at Associated Research Laboratory's Symposium 1977); Personal communication, Croall, G. (1980).
17. Russel, Colin, A., Golay, Noel, G. and Roberts, Gerrylynn, K., *Chemists by Profession: The Origins and Rise of the Royal Institute of Chemistry* (Milton Keynes, 1977), 268.
18. *The Brain Drain, Report of the Working Party on Migration*, Cmnd. 3417 (1967).
19. Murphy, Walter J., *Analytical Chemistry*, **25** (1958), 1431.
20. Lampitt, L. H., *Analyst*, **77** (1952), 568.
21. Association of British Chemical Manufacturers, *Chemical Plant Instrumentation: some Notes on the Use of Measuring Instruments* (Cambridge, 1960), 15. (Hereafter cited as *Measuring Instruments*).
22. *Analysis Instrumentation—1963* (Instrument Society of America, New York, 1963), 20.
23. Vogel, A. I., *A Text Book of Quantitative Inorganic Analysis including Elementary Instrumental Analysis* (London, 1961), v.
24. Williams, F. R., Glenn, A. L. and Jones, A. G., *Proceedings of the Society for Analytical Chemistry*, **2** (1965), 11.
25. *Encyclopaedia Britannica* (1973).
26. *Britannica Book of the Year 1975*, 267.
27. Willard, op. cit., 1727.
28. *Measuring Instruments*, op. cit., 15.
29. Mawson, J., *Automatic Control in the Chemical Process and Allied Industries* (Society of Chemical Industry, London, 1965), 15.
30. Stanley, R., *The Introduction of Automated Analysis in the Chemical and Allied Industries in Britain* (University of Manchester Institute of Science and Technology, 1982).
31. Ibid., 376.
32. Whitehead, S., *Proceedings of the Joint Conference, Institution of Civil of Mechanical and Electrical Engineers* (London, 1951), 473.
33. Smith, E. A., *Mechanising Laboratories* (London, 1965), 13.
34. Personal communications, Baber, J. G. (1980).
35. Beeck, Otto, in *Recent Advances in Analytical Chemistry* (New York, 1949), 69.
36. Croall in Stanley, op. cit., 333.
37. Stanley, op. cit., 332.
38. Ibid., 356.
39. *Encyclopaedia Britannica* (1973).
40. Ibid.
41. Porter, Derrick G., *Automatic Methods of Analysis* (Chelsea College, University of London, 1968).
42. Murphy, Walter J., *Analytical Chemistry*, **25** (1958), 1431.
43. Kuzel, Norbert R., Roudenbush, Harold E. and Stevenson, Charles E., *Journal of Pharmaceutical Sciences*, **58** (1969), 381.
44. Patterson, Gordon D., *Analytical Chemistry*, **29**, 600.
45. Foreman, James K. and Stockwell, Peter B., *Automatic Chemical Analysis* (Chichester, 1975), x.
46. Ibid., ix.
47. Kuzel *et al.*, op. cit., 383.
48. Ibid., 402.
49. Stanley, op. cit., 376.
50. Sargrove, J. A., *Wireless World* (1947).

